# Dnase1L3 Regulates Inflammasome-Dependent Cytokine Secretion

**DOI:** 10.3389/fimmu.2017.00522

**Published:** 2017-05-08

**Authors:** Guilan Shi, Kennady N. Abbott, Wenbo Wu, Russell D. Salter, Peter A. Keyel

**Affiliations:** ^1^Department of Biological Sciences, Texas Tech University, Lubbock, TX, USA; ^2^School of Life Sciences, Lanzhou University, Lanzhou, Gansu, China; ^3^Department of Immunology, University of Pittsburgh, Pittsburgh, PA, USA

**Keywords:** cell activation, inflammation, macrophages, autoimmunity, systemic lupus erythematosus, Dnase gamma

## Abstract

Pediatric-onset systemic lupus erythematosus arises in humans and mice lacking the endonuclease Dnase1L3. When Dnase1L3 is absent, DNA from circulating apoptotic bodies is not cleared, leading to anti-DNA antibody production. Compared to early anti-DNA and anti-chromatin responses, other autoantibody responses and general immune activation in Dnase1L3^−/−^ mice are greatly delayed. We investigated the possibility that immune activation, specifically inflammasome activation, is regulated by Dnase1L3. Here, we report that Dnase1L3 inhibition blocked both NLR family, pyrin domain containing 3 (NLRP3) and NLRC4 inflammasome-mediated release of high-mobility group box 1 protein and IL-1β. In contrast to IL-1β release, Dnase1L3 inhibition only mildly impaired NLRP3-dependent pyroptosis, as measured by propidium iodide uptake or LDH release. Mechanistically, we found that Dnase1L3 was needed to promote apoptosis-associated speck-like protein containing a caspase activation and recruitment domain (ASC) nuclear export and speck formation. Our results demonstrate that Dnase1L3 inhibition separates cytokine secretion from pyroptosis by targeting ASC. These findings suggest that Dnase1L3 is necessary for cytokine secretion following inflammasome activation.

## Introduction

Systemic lupus erythematosus (SLE) is a multifactorial autoimmune disease that affects over one million people in the United States (1). The hallmarks of SLE include chronic inflammation and production of self-reactive antibodies that target nuclear antigens such as anti-double-stranded DNA (anti-dsDNA) antibodies (1, 2). SLE usually manifests in early adulthood and is typically more common in females than in males (1, 3). However, ~10–20% of SLE cases show pediatric onset, where SLE first presents in childhood, sometimes as early as 2 years of age (4, 5). Pediatric-onset SLE is characterized by no gender bias, enhanced lupus nephritis, and central nervous system involvement (4). One genetic cause of pediatric-onset SLE is Dnase1L3 (Dnase γ) deficiency, characterized by anti-dsDNA antibodies, low complement, antineutrophil cytoplasmic antibodies, no gender bias, and a strong incidence of lupus nephritis (5, 6). Partial loss of Dnase1L3 activity in humans can lead to the related autoimmune condition called hypocomplementemic urticarial vasculitis (7, 8), indicating that Dnase1L3 protects humans from autoimmunity.

One model for pediatric-onset SLE is mice deficient in Dnase1L3, which show symptoms similar to humans deficient in Dnase1L3 (6). Dnase1L3^−/−^ mice show early anti-dsDNA and anti-chromatin antibodies, but a delay in overall immune activation (e.g., total IgG production, production of other autoantibodies, and immune-mediated tissue damage) (6). The discrepancy between production of anti-dsDNA antibody and other autoantibodies may be due to the differential availability of autoantigens or due to differential priming of anti-dsDNA antibody responses vs other antibody responses. Anti-dsDNA antibodies can be specifically primed by TLR7 or TLR9. For example, TLR7 and TLR9 promote specific production of anti-dsDNA antibodies in Dnase2a/STING double knockout mice (9). This suggests that Dnase1L3^−/−^ mice could contain a defect in the initial priming steps of their immune response. Indeed, injecting Dnase1L3^−/−^ mice with type I IFN greatly enhances tissue damage, immune responses, and antibody production (6). This suggests that Dnase1L3^−/−^ mice might contain a specific immune activation defect.

Immune activation and inflammation are linked to DNA damage and repair at many levels. On one hand, immune activation typically includes release of reactive oxygen and reactive nitrogen species, which damage DNA (10). Damaged DNA, cytoplasmic DNA, and extracellular DNA all act as danger-associated molecular patterns (DAMPs), which trigger innate immune responses and induce pro-inflammatory cytokine production (10, 11). For example, cytoplasmic DNA is sensed by innate immune sensors STING and AIM2 (11–14). Nucleases that prevent cytoplasmic DNA entry and accumulation, such as Dnase2a in the endosome and Trex1 in the cytoplasm, reduce inflammation (12–14). On the other hand, immune activation can be triggered by DNA repair enzymes such as the Dnase1 superfamily endonuclease apurinic/apyrimidinic endonuclease 1 (APE1) (10). Inactivation of APE1 or other DNA repair pathways reduces pro-inflammatory cytokine production (10). This suggests that DNA-modifying and sensing enzymes are intimately associated with immune activation and pro-inflammatory cytokine production.

Two important pro-inflammatory cytokines are IL-18 and IL-1β. These leaderless cytokines are processed and secreted by the multiprotein complex termed the inflammasome. The inflammasome is composed of a sensory Nod-like receptor (NLR) or PYHIN protein, the adaptor protein apoptosis-associated speck-like protein containing a caspase activation and recruitment domain (ASC), and executioner caspase such as caspase-1 (Casp1), -4, or -11 (15–17). Two of the best studied NLRs are NLR family, pyrin domain containing 3 (NLRP3) and NLR family, caspase activation and recruitment domain containing 4 (NLRC4). NLRP3 recognizes plasma membrane and mitochondrial disruption from a wide range of stimuli, including the bacterial pore-forming toxin (PFT) streptolysin O (SLO) and the potassium ionophore nigericin (15, 16, 18, 19). NLRC4 senses a narrower range of agonists, detecting flagellin and components of type III secretion system from bacteria like *Salmonella enterica* typhimurium (20). Following ligand recognition, NLRs interact with the adaptor ASC (15, 16). To interact with most NLRs, ASC must translocate to the cytosol from the nucleus (21). Which signals induce ASC nuclear egress remain unknown, although IKKα degradation is one of the steps in the pathway (22). Once in the cytosol, ASC recruits Casp1 and forms a prion-like structure termed either pyroptosome or ASC speck (23–25). ASC specks oligomerize Casp1 (23–25). Casp1 oligomerization induces autoproteolysis, cleaving the Casp1 p45 zymogen into active p20 and p10 subunits (15, 16). Active Casp1 directly cleaves pro-IL-1β and pro-IL-18 to their mature forms. Casp1 also activates the endogenous PFT gasdermin D, which leads to cell lysis termed pyroptosis (26–29). NLRP3 requires ASC for Casp1 interaction, though NLRC4 can directly interact with Casp1 (30, 31). However, ASC is needed for full cytokine production following NLRC4 activation (30–32). *In vitro* inflammasome activation and IL-1β release can be triggered in two steps, termed priming and activation (16). Macrophages, such as primary murine bone marrow-derived macrophages (BMDM), are primed with a TLR ligand such as lipopolysaccharide (LPS), which activates NF-KB signaling and upregulation of inflammasome components and triggers pro-IL-1β synthesis (16, 17, 33). Once primed, macrophages are stimulated with the NLR ligand and inflammasome activation is assessed.

Along with cytokines, inflammasome activation releases DAMPs like high-mobility group box 1 protein (HMGB1) (34, 35). HMGB1 is an abundant non-histone nuclear transcription factor that lacks secretion signals (36). Following 24 h treatment with LPS, type I IFN production promotes HMGB1 export to the cytosol through Janus kinase signaling (37). During necrosis or other forms of cell lysis, HMGB1 can also be passively released from the cell (36). Once released from the cell, HMGB1 acts as a late-phase mediator of lethal endotoxic shock and sterile injury (38). The mechanism through which the inflammasome secretes HMGB1 remains unknown. However, HMGB1 release during apoptosis is blocked by Dnase1L3 inhibition (39). Three Dnase1L3 inhibitors are known: fmoc-d-cyclohexylalanine (FCA), pontacyl violet 6R (PV), and DR396 (39). While DR396 is considered the most potent (39), it is not commercially available. These inhibitors are useful tools for evaluating whether there is a role for Dnase1L3 during inflammasome activation.

Dnase1L3 is a Ca^2+^/Mg^2+^-dependent endonuclease in the Dnase superfamily and closely related to Dnase1. In contrast to Dnase1, Dnase1L3 is expressed predominantly in myeloid cells such as macrophages (6). It is most active at neutral pH, leaves 5′ phosphates following DNA cleavage, and has a greater affinity for cleaving chromatin and nucleosomes than naked DNA (40, 41). Along with chromatin, Dnase1L3 also cleaves apoptotic bodies and microparticles, allowing it to act as a barrier to transfection (6, 42). The barrier to transfection activity is mediated through a helical *C*-terminus *via* an unknown mechanism (6, 42). Mutations that reduce either nuclease activity, like R206C, or barrier to transfection activity are associated with autoimmunity (7, 8). This indicates that Dnase1L3 has an important enzymatic activity.

The localization of Dnase1L3 is controversial. It has a signal peptide that directs secretion (40, 43). Extracellularly, Dnase1L3 provides barrier to transfection and protection from pediatric-onset SLE (6, 42). However, Dnase1L3 relocalizes to the nucleus when the signal sequence is missing, presumably due to the two nuclear localization sequences in Dnase1L3 (44–46). In the nucleus, Dnase1L3 degrades DNA during apoptosis in a variety of cell lines (41, 44). Further evidence for an intracellular role is the necessity of Dnase1L3 for induction of apoptosis by acetaminophen and chemotherapeutic agents (47, 48). During apoptosis, Dnase1L3 facilitates internucleosomal cleavage (41). Whether two pools of Dnase1L3 exist or whether Dnase1L3 is relocalized is unknown, although it is clear that Dnase1L3 can act both extracellularly and intracellularly.

In the present study, we tested the hypothesis that Dnase1L3 regulates inflammasome activation. We found that Dnase1L3 inhibition using either FCA or PV potently blocked IL-1β processing and release following NLRP3 inflammasome stimulation without directly inhibiting Casp1 or blocking TNFα release. In contrast, HMGB1 release was ~50% inhibited by FCA under conditions that allowed no IL-1β release, suggesting that unlike IL-1β, HMGB1 can be partially released by pyroptosis. Indeed, pyroptosis was only mildly impaired in these cells. FCA may be a general inflammasome inhibitor, since IL-1β processing by the NLRC4 inflammasome was also blocked by Dnase1L3 inhibition. Dnase1L3 RNA interference (RNAi) confirmed that the blockade observed with inhibitor treatment was specific to Dnase1L3 inhibition. Mechanistically, we found that FCA blocked ASC nuclear export and speck formation. Taken together, these data suggest that Dnase1L3 is necessary for inflammasome-mediated IL-1β processing and that Dnase1L3 inhibition uncouples cytokine secretion from pyroptosis. Overall, we propose a mechanistic basis for the delay between anti-dsDNA antibody production and other immune activation observed in the murine model of pediatric-onset SLE.

## Materials and Methods

### Reagents

Unless noted, all reagents were from Thermo Fisher Scientific (Waltham, MA, USA). FCA, nigericin, and Ponceau S were from Sigma-Aldrich (St. Louis, MO, USA). PV was from TCI America (Portland, OR, USA). Ac-YVAD-cmk was from Enzo Life Sciences (Farmingdale, NY, USA). Fam-YVAD-fmk (FLICA) was from ImmunoChemistry Technologies (Bloomington, MN, USA). Ultrapure LPS was from Invivogen (San Diego, CA, USA). One endotoxin unit per milliliter ultrapure LPS is approximately equal to 1 ng/mL from the same manufacturer. GelRed was from Phenix Research Products (Candler, NC, USA). SLO was purified, and hemolytic activity was assessed as previously described (49). The specific activity of SLO was 1.07 × 10^6^ HU/mg. Murine IL-1β cloned into pFB-Neo retroviral vector (Agilent, Santa Clara, CA, USA) using *Not*I and *Eco*RI was obtained from Dr. Chenqun Sun (University of Pittsburgh). Murine Casp1 was cloned into peCFP-N1 (Clontech, Mountain View, CA, USA) using *Xho*I and *Not*I, which removed the fluorescent protein. Human Dnase1L3 was cloned into pIRES-DsRed (Clontech) using *Eco*RI and *Bam*HI sites. The R206C mutation was made in Dnase1L3 *via* Quikchange mutagenesis (Agilent, Santa Clara, CA, USA). PCR primers were from Integrated DNA Technologies (Coralville, IA, USA) with sequences available upon request.

### Antibodies

The anti-HMGB1 mouse monoclonal antibody (mAb, Catalog: ab79832) was from Abcam (Cambridge, MA, USA). The anti-β-actin mAb AC-15 (Catalog: 612657) was from BD Biosciences (Franklin Lakes, NJ, USA). The anti-IL-1β mAb 3ZD was from the Frederick National Laboratory for Cancer Research (Frederick, MD, USA) (50). The anti-Casp1 mAb Casper-1 (Catalog: AG-20B-0042-C100) was from AdipoGen (San Diego, CA, USA). Anti-ASC mAb TMS-1 (Catalog: 653902), capture and detection anti-TNFα antibodies (Catalog 79092 and 78335, respectively), capture and detection anti-human IL-1β antibodies (Catalog: 508302 and 508202, respectively), streptavidin conjugated to HRP (Catalog 405210), and TMB substrate (Catalog 421101) were from BioLegend (San Diego, CA, USA). Capture and detection anti-murine IL-1β antibodies (Catalog: 14-7012 and 13-7112, respectively) were from eBioscience (San Diego, CA, USA). Anti-Lamin A/C (MANLAC-4A7-s) was deposited by G.E. Morris (DSHB Hybridoma Product MANLAC1(4A7)) to the Developmental Studies Hybridoma Bank, which was created by the NICHD of the NIH and maintained at The University of Iowa, Department of Biology, Iowa City, IA, USA. Fluorescently conjugated secondary antibodies (Catalog: M31501) were from Thermo Fisher Scientific. HRP-conjugated secondary antibodies (anti-mouse 715-035-151; anti-rabbit 711-035-152) were from Jackson Immunoresearch (West Grove, PA, USA).

### Mice

All mice were housed and maintained at the University of Pittsburgh or Texas Tech University according to respective IACUC standards. Bone marrow from NLRP3^−/−^ mice was a generous gift from Dr. Tim Billiar, while bone marrow from Casp1^−/−^ mice was a generous gift from Dr. Richard Flavell, and bone marrow from C57BL/6 mice was a generous gift from Dr. Lisa Borghesi. Additional mice were purchased from The Jackson Laboratory (stock # 000664, 021302, and 016621 for C57BL/6, NLRP3^−/−^, and Casp1^−/−^, respectively). Mice of both gender aged 6–15 weeks were used to prepare BMDM. The source of the bone marrow did not alter the results. Sample size was determined as the minimum number of mice needed to provide sufficient bone marrow for the experiments. Consequently, no randomization or blinding was needed.

### Cells

Bone marrow-derived macrophages (BMDM) were isolated and cultured as previously described (19, 33). THP1 cells (ATCC TIB-202) were cultured at 37°C in DMEM supplemented with 10% FCS and 1× l-glutamine (D10) and 1× penicillin/streptomycin and differentiated for 2 days using 5 ng/mL phorbol 12-myristate 13-acetate (PMA) prior to assay. HEK cells (ATCC CRL-1573) were cultured in D10 at 37°C. All cell lines were negative for mycoplasma.

### Transfections

THP1 cells were transfected using Lipofectamine RNAiMAX and 10 pM siRNA (Dnase1L3 HSS 102839) pools directed against Dnase1L3 or control siRNAs (Catalog: 1691553) for 2 days in the presence of 5 ng/mL PMA. Pools of multiple Dnase1L3 siRNAs were used instead of individual siRNAs to reduce the potential for off-target effects (51). RNAi efficiency was evaluated by comparing mRNA expression and phenotype between specific Dnase1L3 siRNAs and non-specific, control siRNAs, in accordance with previously accepted RNAi guidelines (52). HEK cells were plated at 2.5 × 10^5^ cells/well 1 day prior to transfection with 500 ng of indicated plasmids using Lipofectamine2000 and incubated at 37°C either overnight (IL-1β/Casp1) in the presence of nothing, 100 µM FCA, or 20 µM PV for 2 days (Dnase1L3) at 37°C at which point in time supernatants were collected for ELISA or cell lysates generated.

### RT-qPCR

Total RNA was isolated using TriReagent, according to the manufacturer’s instructions. RNA was used for cDNA synthesis with SuperScript^®^ III reagents. Quantitative PCR on duplicate samples was performed using the SYBR^®^ Select Master Mix and normalized to the housekeeping gene GAPDH. Primers used were 5′-GATCATCAGCAATGCCTCCT-3′ and 5′-TGTGGTCATGAGTCCTTCCA-3′ for human GAPDH; 5′-AGCCCTTTGTGGTCTGGTTC-3′ and 5′-TCCTTAACGGATGTCTCTGGG-3′ for human Dnase1L3; 5′-GAAATCGTGCGTGACATCAAAG-3′. Relative expression of Dnase1L3 was calculated using the ΔΔCt standardization method.

### Western Blotting

Supernatants were trichloroacetic acid (TCA) precipitated (50), and cells were lysed in SDS sample buffer, denatured, boiled for 5 min, resolved by SDS-PAGE, and proteins transferred onto a 0.45-µm nitrocellulose membrane. Immunoblotting with primary antibodies Casper-1 anti-Casp1 (1:3,000), 3ZD anti-IL-1β (1:1,000), EPR3057 anti-HMGB1 (1:3,000), MANLAC1 anti-Lamin A/C (1:1,000), TMS-1 anti-ASC (1:1,000), and AC-15 anti-β-actin (1:3,000) was followed by secondary anti-rabbit or anti-mouse antibodies conjugated to HRP (1:10,000). Antibody staining was visualized on a FluorChemE (Protein Simple, San Jose, CA, USA) using enhanced chemiluminescence reagent [0.01% H_2_O_2_ (Walmart, Bentonville, AR, USA), 0.2 mM p-Coumaric acid (Sigma-Aldrich), 1.25 mM Luminol (Sigma-Aldrich), and 0.1 M Tris, pH 8.4]. Immunoblots were analyzed using Photoshop Creative Suite 3 (Adobe, San Jose, CA, USA). The images were inverted, and integrated intensity of the bands was measured. The percentage of IL-1β or HMGB1 in the supernatant was ratioed to pro-IL-1β or HMGB1 in the lysate and then expressed as a percentage of the control. Pro-IL-1β and lysate HMGB1 levels were used because the actin loading control was saturated in many experiments. In those experiments where actin was not saturating, normalizing to actin instead of pro-IL-1β or HMGB1 did not alter the results.

### Inflammasome Activation

BMDM (10^5^ for ELISA or immunofluorescence and 10^6^ for immunoblot) were primed with 100 EU/mL ultrapure LPS for 4 h, washed in PBS, and stimulated with 20 µM nigericin or 0.5 kHU/mL SLO for 0–30 min in RPMI at 37°C. This SLO dose is sublytic in macrophages (19). BMDM were incubated with inhibitors for 30 min prior to and during inflammasome stimulation at the following concentrations: 100 µM FCA, 20 µM PV, 50 mM KCl, and 100 µM YVAD. Inhibitor stock solutions were 10 mM FCA in ethanol, 10 mM PV in water, 4 M KCl in water, and 50 mM YVAD in DMSO. Equivalent volumes of ethanol or DMSO had no effect in any experiments performed. For *Salmonella enterica* typhimurium infection, BMDM were LPS primed for 4 h prior to infection at an MOI of 50 for 1 h. BMDM were incubated for 1 h with FLICA in some experiments along with either SLO or nigericin. The additional time was necessary since FLICA is based on YVAD, which binds to the active site of Casp1 and slows inflammasome activation at the doses of FLICA used.

### Immunofluorescence

Cells were fixed for 15 min in 2% paraformaldehyde; permeabilized/blocked for 15 min in 0.1% Triton X-100, 10% goat serum, and 0.05% saponin; stained with EPR3057 anti-HMGB1 mAb or TMS-1 anti-ASC mAb for 1 h, followed by goat anti-rabbit-Alexa 488 or goat anti-rabbit-Cy5 and goat anti-mouse Cy3 for 1 h, and finally DAPI stained. Cells were imaged by epifluorescence microscopy using either an Olympus Provis equipped with 20× (0.85 NA) or 60× (1.42 NA) objective lenses running Magnafire 2.1 (Olympus, Center Valley, PA, USA) or an Olympus BX41 equipped with 60× (1.40 NA) objective running QCapture Pro 7 (QImaging, Surrey, BC, Canada). Nuclear HMGB1 egress was calculated using Metamorph (Molecular Devices, Sunnyvale, CA, USA) to determine the average nuclear intensity of HMGB1. HMGB1 low cells were determined to be those cells containing less nuclear fluorescence intensity than 80–90% of untreated cells in the same experiment. The background levels of untreated cells were then subtracted from each condition. ASC speck formation in THP1 cells was quantitated using ImageJ (53).

### ELISA

Cytokine levels in cell culture supernatants were determined by ELISA. TNFα ELISA was performed according to the manufacturers’ instructions. IL-1β ELISA was performed as previously described (19, 33) although murine IL-1β capture and detection antibody concentrations were 2 and 3 µg/mL, respectively, and human IL-1β capture and detection antibody concentrations were 3 and 2 µg/mL, respectively.

### Dnase Assay

HEK cells were grown for 24 h at 2.5 × 10^5^ cells per well. The cells were transfected with Dnase1L3 or Dnase1L3 R206C or left untreated and incubated at 37°C, 5% CO_2_ for 24 h. The cells were harvested with trypsin, centrifuged at room temperature for 5 min at 1,000 × *g*, washed with PBS, and centrifuged again. The cell pellets were resuspended in Dnase assay buffer (20 mM Tris, pH 7.4, 5 mM MgCl_2_, 2 mM CaCl_2_) with 1% Triton X-100 and 0.5% phenylmethylsulfonyl fluoride and then kept on ice for 10 min. The cells were then centrifuged at 4°C for 15 min at 17,000 × *g*. The supernatants were saved for Dnase activity assays. Dnase1L3 activity was determined by incubating 400 ng plasmid DNA with cell lysates in the presence or absence of 50 µM FCA, PV, or aurintricarboxylic acid (ATA) in 20 mM Tris, pH 7.4, 5 mM MgCl_2_, 2 mM CaCl_2_ for 30 min at 37°C. The extent of degradation was quantitated using Photoshop Creative Suite 3 from Gel Red-stained agarose gels. Transfection efficiency was determined by flow cytometry, and equal transfection was observed between Dnase1L3 wild type (WT) and R206C. Dnase1L3 activity was determined by titrating lysates until 50–70% degradation was observed. This dilution was then used to determine inhibitor activity.

### Pyroptosis

BMDM were primed for 4 h at 37°C with 100 ng/mL LPS, harvested, incubated with various concentrations of SLO in RPMI with 20 µg/mL propidium iodide (PI) for 5 or 30 min at 37°C and analyzed by FACS on an LSR II (BD Biosciences). Debris, defined as any event with a subcellular forward and side scatter profile, was gated out, and the percentage of cells in the PI-high gate was determined. Specific lysis was determined by subtracting the background from each point and dividing by 100 less the background. LDH assays were performed according to manufacturer’s instructions.

### Annexin V Assay

BMDM (10^6^) were treated with SLO at various concentrations for 30 min at 37°C or UV irradiated for 15 s followed by 4 h culture at 37°C. THP1 (5 × 10^5^) cells were either treated with SLO at various concentrations for the indicated time points or treated with 15 µM etoposide (Etop) for 6 h at 37°C. Following treatment, cells were stained with 0.9 µg/mL Annexin V-FITC and 20 µg/mL PI in Annexin V Binding Buffer (1.4 mM NaCl, 25 mM CaCl_2_, 100 mM HEPES, pH 7.4) for 15 min on ice and analyzed by FACS on an Accuri C6 (BD Biosciences). Debris, defined as any event with a subcellular forward and side scatter profile, was gated out, and the percentage of PI^+^, Annexin V^+^, or double-positive cells was determined.

### Data Presentation and Statistical Analysis

Prism 5.0 (GraphPad, La Jolla, CA, USA) software was used for statistical analysis. Data are represented as mean ± SEM as indicated. Statistical significance was determined by one-way or two-way ANOVA with Bonferroni posttesting; *p* < 0.05 was considered statistically significant.

## Results

### Dnase1L3 Regulates IL-1β Release

**Figure 1:**
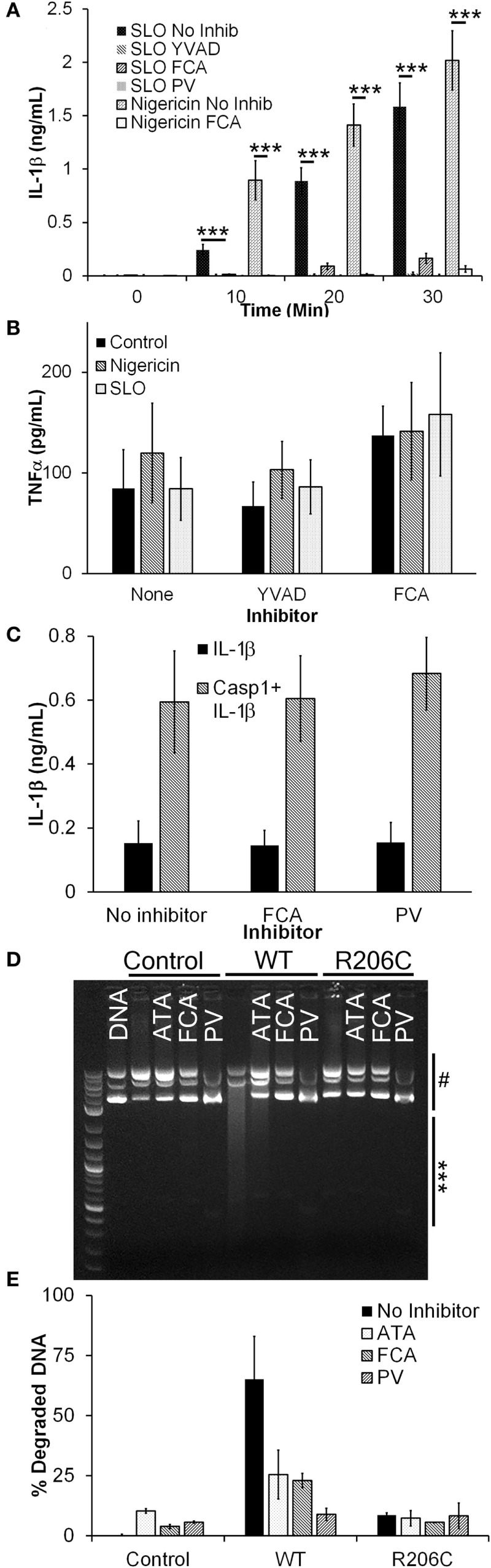
**Dnase1L3 inhibitors block IL-1β release**. **(A,B)** Lipopolysaccharide-primed BMDM were treated with the indicated inhibitor for 30 min followed by either 0.5 kHU/mL streptolysin O (SLO) or 20 µM nigericin for either the indicated times **(A)** or 30 min **(B)**, and supernatants assayed for IL-1β **(A)** or TNFα **(B)** by ELISA. **(C)** HEK cells were transfected with IL-1β with or without caspase-1 (Casp1) in the presence of no inhibitor, 100 µM fmoc-d-cyclohexylalanine (FCA), or 10 µM pontacyl violet 6R (PV) overnight and IL-1β levels in the supernatants measured by ELISA. **(D,E)** HEK cells were untransfected (control) or transfected with Dnase1L3 [wild type (WT)] or Dnase1L3 R206C (R206C), lysed, and Dnase1L3 activity measured. Sufficient WT lysate to promote 50–70% plasmid DNA degradation or an equivalent amount of mock or R206C lysate was incubated in the presence or absence of 50 µM FCA, PV, or aurintricarboxylic acid (ATA) for 30 min at 37°C. DNA degradation was determined by measuring integrated intensity of degraded (***) and intact (^#^) plasmid DNA following gel electrophoresis and calculating the percentage of degraded DNA. The gel is representative of two independent experiments. The graphs represent mean ± SEM of at least five **(A)**, six **(B)**, four **(C)**, or two **(E)** independent experiments. ***p < 0.001.

We investigated whether Dnase1L3 was needed for IL-1β secretion in B6 BMDM using Dnase1L3 inhibitors. We tested whether the Dnase1L3 inhibitors, FCA and PV, blocked inflammasome-dependent IL-1β production. To trigger inflammasome activation, we used either the prototypical NLRP3 agonist, nigericin, or the more biologically relevant, tunable NLRP3 agonist, SLO. We treated LPS-primed BMDM with either sublytic SLO or nigericin and measured IL-1β secretion in the absence or presence of FCA, PV, or the Casp1 inhibitor YVAD. Similar to YVAD-mediated inhibition, both FCA and PV potently blocked IL-1β secretion (Figure 1A). Since FCA blocked IL-1β release, it is possible that FCA generally blocked cytokine release. To test this hypothesis, we next measured the levels of TNFα secreted by the LPS-primed BMDM following inhibitor treatment during inflammasome activation. We found that NLRP3 inflammasome activation did not reduce TNFα secretion (Figure 1B). Furthermore, treatment with either YVAD or FCA did not reduce TNFα levels (Figure 1B). These findings demonstrate that inhibition of Dnase1L3 does not block general cytokine release but blocks IL-1β release in primary B6 BMDM stimulated with NLRP3 inflammasome activators.

The IL-1β blockade we observed by FCA or PV treatment could be due to direct inhibition of Casp1 *via* inhibitor off-target effects. To test this hypothesis, we transfected HEK cells with IL-1β and Casp1. When Casp1 is overexpressed in HEK cells, it autoactivates without NLRs or ASC and processes any pro-IL-1β present in the cells (54, 55). When we treated transfected HEK cells with either FCA or PV, we found no change in IL-1β secretion (Figure 1C). These results indicate that Casp1 is not a direct target of these Dnase1L3 inhibitors.

We next confirmed that FCA and PV inhibit Dnase1L3 activity by expressing either WT or R206C Dnase1L3 in HEK cells. The R206C mutation eliminates Dnase1L3 activity (8). To control for transfection efficiency between experiments, we titrated the Dnase1L3 activity to degrade ~60% of a fixed amount of plasmid DNA. Transfected Dnase1L3 was the only source of Dnase1L3 activity in HEK lysates, and this activity was eliminated by the R206C mutation (Figures 1D,E). When lysates were incubated with either FCA or PV, we observed a reduction in Dnase1L3 activity similar to that provided by the R206C mutation (Figures 1D,E). The inhibition was also similar to that observed with the general nuclease inhibitor ATA (Figures 1D,E). These findings suggest that FCA and PV block Dnase1L3 activity.

### Sublytic SLO Induces NLRP3 Inflammasome Activation and HMGB1 Release

**Figure 2:**
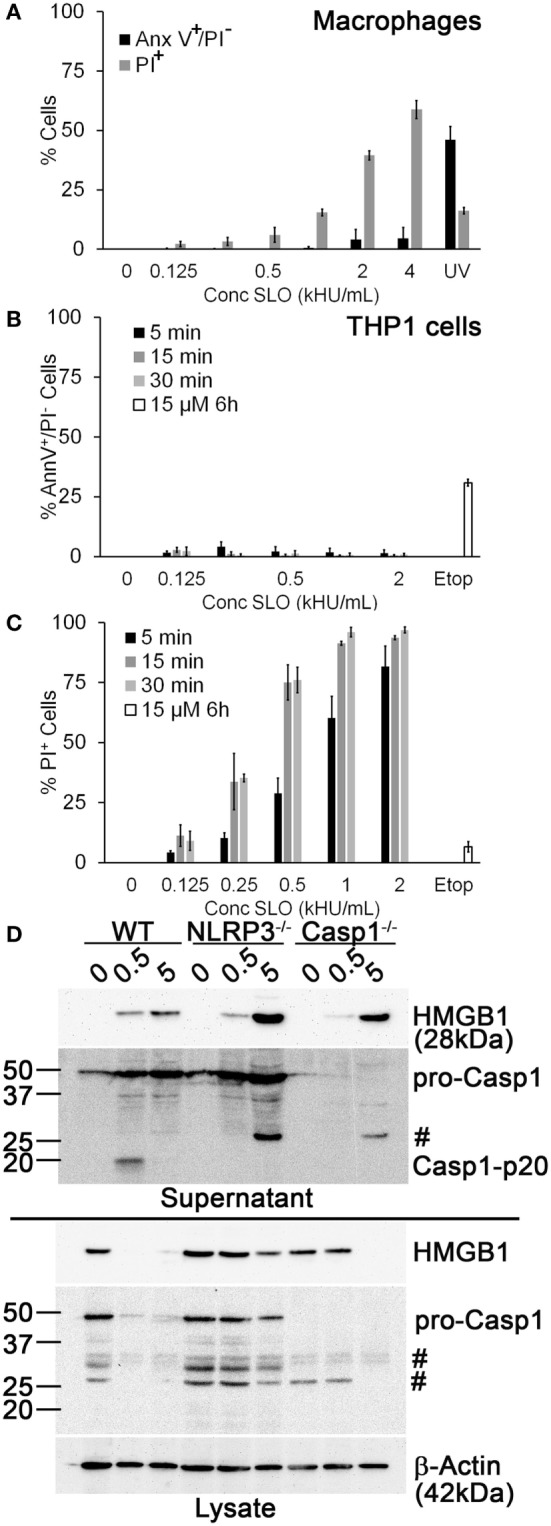
**Sublytic streptolysin O (SLO) promotes NLR family, pyrin domain containing 3 (NLRP3) inflammasome-dependent high-mobility group box 1 protein (HMGB1) release**. **(A)** BMDM from C57BL/6 [wild-type (WT)] mice were treated with SLO at different concentrations (kHU/mL) for 30 min or UV irradiated for 15 s followed by 4 h culture, stained with 0.9 µg/mL Annexin V-FITC and 20 µg/mL propidium iodide (PI), and analyzed by FACS. **(B,C)** THP1 cells were either treated with SLO at different concentrations for the indicated time points or treated with 15 µM etoposide (Etop) for 6 h, then stained with 0.9 µg/mL Annexin V-FITC and 20 µg/mL PI, and analyzed by FACS. The percentage of apoptotic (PI^−^/Annexin V^+^) **(B)** or necrotic (PI^+^) **(C)** cells are shown. **(D)** Lipopolysaccharide-primed BMDM from WT, Casp1^−/−^, or NLRP3^−/−^ mice were treated with SLO at sublytic (0.5 kHU/mL) or lytic (5 kHU/mL) concentrations for 30 min. Supernatants were trichloroacetic acid precipitated, and cells were lysed in SDS sample buffer. Proteins were resolved by SDS-PAGE and transferred to nitrocellulose. HMGB1, caspase-1 (Casp1), and β-actin were detected by western blot. The graphs represent mean ± SEM of three independent experiments. The immunoblot is representative of four independent experiments. ^#^Non-specific bands.

Another potential explanation for our finding that Dnase1L3 inhibition blocked IL-1β release is that the inhibitor targets SLO-dependent cell death cells independently of inflammasome activation prior to IL-1β release (19). Since SLO challenge is associated with pyroptosis, necrosis, and apoptosis (18, 19, 56), we determined the dose of SLO needed to kill human THP1 cells and primary B6 BMDM and the mechanism of cell death. We measured necrosis using PI uptake and simultaneously we measured apoptosis using Annexin V. Following 30-min SLO treatment, we did not detect any apoptosis in BMDM or THP1 cells (Figures 2A,B). In contrast, UV irradiation or etoposide treatment for longer periods of time promoted apoptosis in BMDM and THP1 cells (Figures 2A,B). Consistent with previous results (19), we found that unprimed BMDM were more resistant to lysis by SLO than other cell types, including THP1 cells (Figures 2A,C). For BMDM, 0.5 kHU/mL was determined to be a sublytic dose, while 4–5 kHU/mL provided a lytic dose (Figure 2A). In contrast, the sublytic dose for THP1 cells was found to be 0.125 kHU/mL and lytic dose to be 0.5 kHU/mL (Figure 2C). Thus, SLO promoted neither apoptosis nor necrosis at sublytic concentrations.

We tested whether sublytic SLO challenge promotes inflammasome activation by comparing Casp1 activation and HMGB1 secretion in WT, NLRP3^−/−^, and Casp1^−/−^ BMDM treated with sublytic or lytic doses of SLO. We found that in WT BMDM, sublytic SLO doses activated Casp1 and depleted HMGB1 from cell lysates, resulting in extracellular HMGB1 release (Figure 2D). In contrast, lytic SLO doses released HMGB1 without Casp1 activation (Figure 2D). In NLRP3^−/−^ and Casp1^−/−^ BMDM, sublytic SLO promoted minor HMGB1 egress, without appreciably depleting cytoplasmic stores (Figure 2D). These results indicate that robust HMGB1 egress under these conditions requires inflammasome activation. In contrast, lytic SLO doses promoted HMGB1 release independently of NLRP3 or Casp1 activation (Figure 2D). Thus, sublytic SLO can promote HMGB1 release and Casp1 activation in a NLRP3 inflammasome-dependent manner. Furthermore, SLO dose does not account for the FCA- or PV-mediated IL-1β blockade.

### Dnase1L3 Inhibition Impairs HMGB1 Release

**Figure 3:**
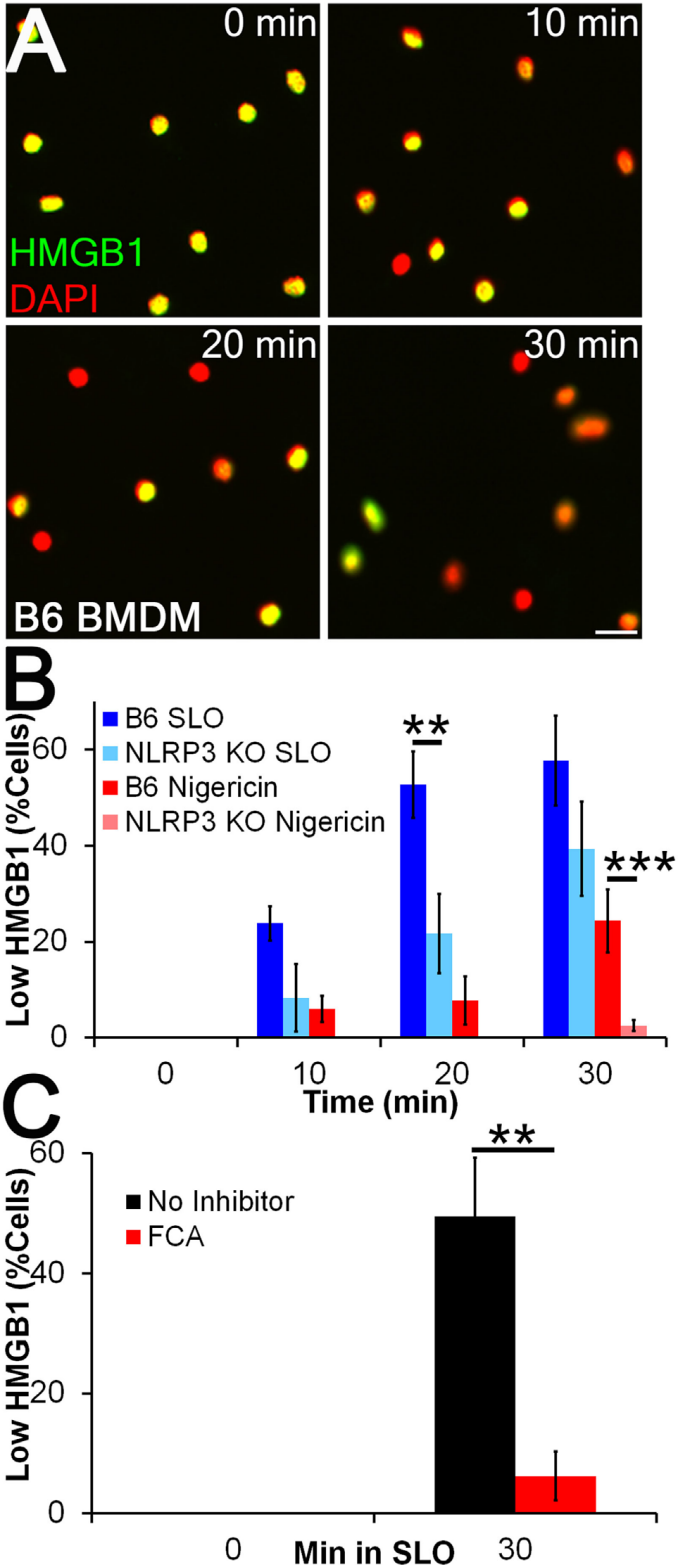
**Inflammasome-dependent high-mobility group box 1 protein (HMGB1) release is impaired by Dnase1L3 inhibitors**. **(A,B)** BMDM from wild type or NLRP3^−/−^ mice were primed for 4 h with 100 EU/mL lipopolysaccharide (LPS) at 37°C and treated with 20 µM nigericin **(A, B)** or 0.5 kHU/mL streptolysin O (SLO) **(B)** for the indicated times. Cells were fixed and stained for HMGB1 (green) and DAPI (red). The percentage of cells showing HMGB1 nuclear egress (Low HMGB1) was quantitated in Metamorph. **(C)** LPS-primed BMDM were treated with 100 µM fmoc-d-cyclohexylalanine (FCA) for 30 min followed by 0.5 kHU/mL SLO for 30 min, fixed and stained for HMGB1 and DAPI. The percentage of cells showing HMGB1 nuclear egress (low HMGB1) is shown. Micrographs are representative of four independent experiments. The graphs represent mean ± SEM of four and eight for nigericin and SLO, respectively, **(B)** or nine **(C)** independent experiments. **p < 0.01, ***p < 0.001. Scale bar = 20 µm.

We next tested whether FCA blocks HMGB1 release. Since nuclear egress seemed to be a more sensitive readout of inflammasome-dependent activation than presence of HMGB1 in the supernatant (Figure 2D), we measured nuclear HMGB1 levels by immunofluorescence. We treated LPS-primed BMDM from WT and NLRP3^−/−^ mice with sublytic SLO or nigericin for 10–30 min and measured HMGB1 release (Figures 3A,B). We found that 4 h of LPS priming was insufficient to promote HMGB1 export, as previously described (35), but that subsequent sublytic SLO or nigericin treatment rapidly promoted the release of nuclear HMGB1 (Figures 3A,B). We found that HMGB1 egress was reduced in NLRP3^−/−^ BMDM, indicating HMGB1 release under these conditions was NLRP3 dependent (Figure 3B). We next used this system to determine whether Dnase1L3 contributes to inflammasome-dependent HMGB1 release during toxin challenge. We treated LPS-primed BMDM with 100 µM FCA followed by sublytic SLO challenge and measured HMGB1 nuclear egress (Figure 3C). Compared to the control, the level of HMGB1 release from FCA-treated cells was significantly reduced (Figure 3C). Thus, robust HMGB1 release following NLRP3 inflammasome activation requires Dnase1L3 nuclease activity.

### Dnase1L3 Inhibition Blocks Casp1 Cleavage and IL-1β Processing

**Figure 4:**
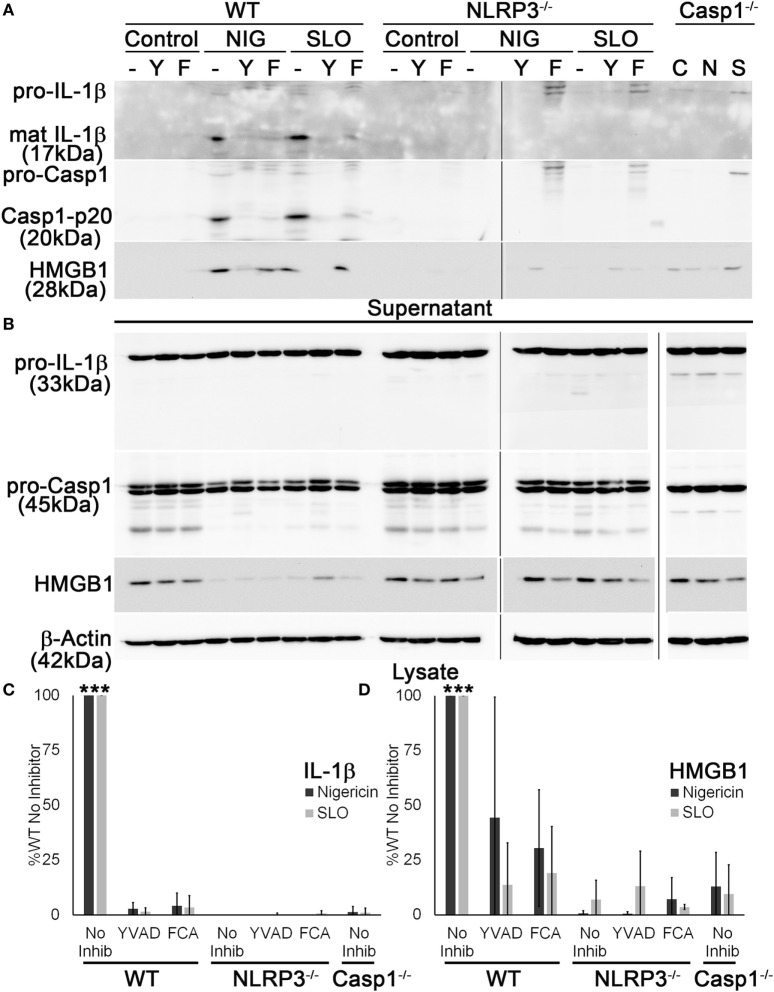
**Dnase1L3 activity is required for caspase-1 (Casp1) activation**. Lipopolysaccharide-primed BMDM from wild-type (WT), Casp1^−/−^, or NLRP3^−/−^ mice were treated without (−) or with 100 µM YVAD (Y) or 100 µM fmoc-d-cyclohexylalanine (FCA) (F) for 30 min followed by nothing (C), 20 µM nigericin (N or NIG), or 0.5 kHU/mL streptolysin O (SLO) (S) for 30 min. Supernatants **(A)** were trichloroacetic acid precipitated, and cells **(B)** were lysed in SDS sample buffer. Proteins were resolved by SDS-PAGE and transferred to nitrocellulose. High-mobility group box 1 protein (HMGB1), Casp1, IL-1β, and β-actin were detected by western blot. **(C)** The levels of mature IL-1β in the supernatant were ratioed to pro-IL-1β levels in the cell lysates, or **(D)** levels of secreted HMGB1 were ratioed to HMGB1 in the lysate, and all expressed as a percentage of B6 BMDM treated with no inhibitor. Immunoblots are representative of four independent experiments, while graphs show mean ± SEM of four independent experiments. ***p < 0.001 compared to inhibitors and knockouts (except compared to WT YVAD for HMGB1, which was not significant).

Since it appeared that FCA blockade reduced both IL-1β secretion and HMGB1 release, we next tested whether Dnase1L3 is needed for Casp1 cleavage. We treated WT, Casp1^−/−^, or NLRP3^−/−^ LPS-primed BMDM with sublytic SLO or nigericin with or without YVAD or FCA and examined processing and release of IL-1β and Casp1. Mature IL-1β and Casp1 p20 were detected in the TCA-precipitated supernatant of WT BMDM (Figure 4A). In contrast, both FCA and YVAD reduced mature IL-1β or active Casp1 levels to that observed in Casp1^−/−^ and NLRP3^−/−^ BMDM (Figure 4A). There was slight, variable toxicity to the cells from FCA, as measured by pro-IL-1β release in FCA-treated knockout BMDM and a slight (but not statistically significant) decrease in lysate levels of actin and other proteins in the cell lysate in WT, NLRP3^−/−^, and Casp1^−/−^ BMDM (Figure 4B). Although IL-1β levels were decreased tremendously following inhibitor treatment or in the absence of NLRP3 or Casp1, HMGB1 egress was reduced ~50% following treatment with YVAD or FCA (Figures 4C,D). This impairment was somewhat variable, though generally ~50%. The incomplete inhibition of HMGB1 release by these assays could be due to cell lysis. Overall, we find that Dnase1L3 activity is required for IL-1β and Casp1 processing.

### Dnase1L3 Inhibition Only Mildly Impairs Pyroptosis

**Figure 5:**
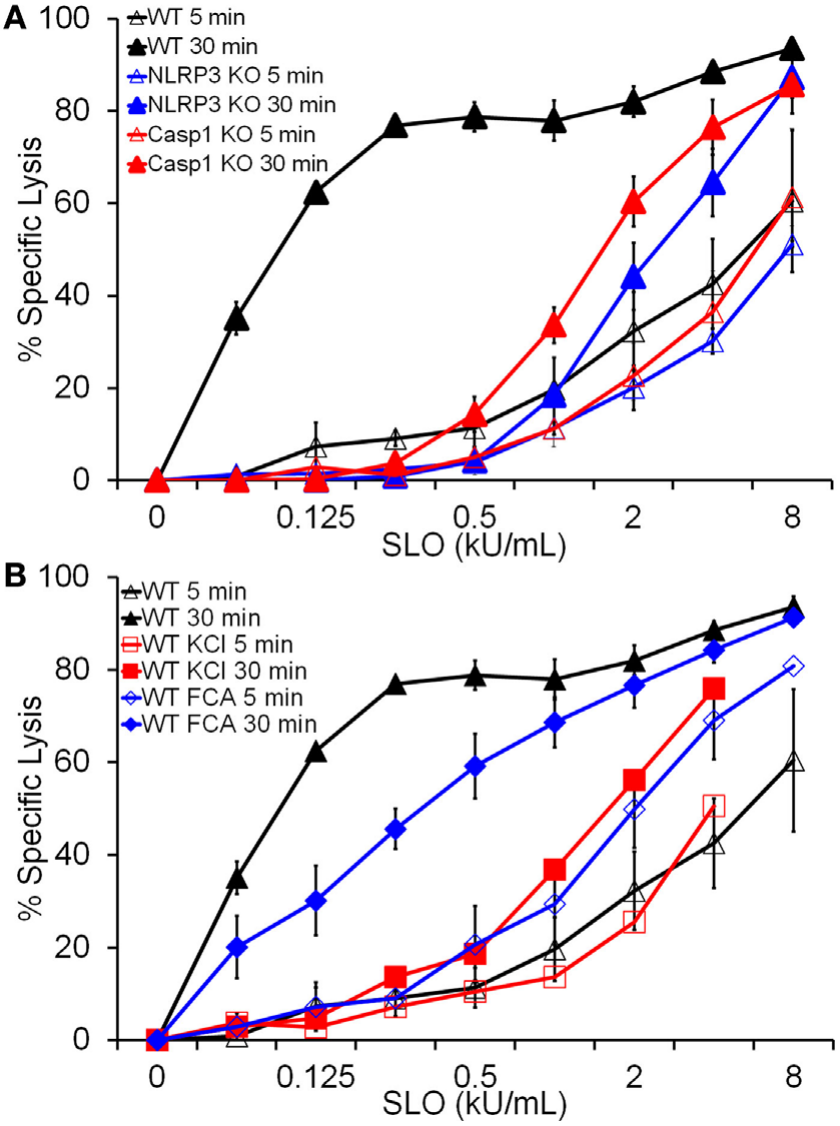
**Dnase1L3 inhibition mildly impairs pyroptosis**. Lipopolysaccharide-primed BMDM of the indicated genotypes were treated with the indicated inhibitors and streptolysin O (SLO) at the listed concentrations for 5 or 30 min in the presence of propidium iodide and analyzed *via* FACS. The same data for wild-type BMDM samples were used for both panels **(A,B)**. Graphs are mean ± SEM of at least three independent experiments.

Since HMGB1 is released by cell lysis (Figure 2D), it is possible that FCA does not significantly impair pyroptosis. We next tested whether Dnase1L3 is required for pyroptosis. Pyroptosis typically occurs after 30 min of nigericin or SLO treatment, kinetically similar to IL-1β release (19) and is usually measured *via* membrane integrity assays in the presence and absence of inflammasome inhibitors or in Casp1/11 knockout cells. For consistency with previous results (19), we chose to use SLO to measure pyroptosis. Since lysis due to SLO is also measured *via* membrane integrity, we took advantage of the kinetic differences between direct SLO lysis and pyroptosis to distinguish between pyroptosis and direct SLO-mediated lysis. We treated LPS-primed BMDM with SLO for 5 min to assess direct SLO cytotoxicity or 30 min to allow time for pyroptosis. We measured specific cell lysis by PI uptake. FCA increased baseline sensitivity from 21% ± 3.79 (mean ± SEM) in control BMDM to 35.3% ± 1.65 (*p* = 0.055) at 30 min, indicating slight toxicity to cells from FCA. When challenged with SLO, WT, NLRP3^−/−^, and Casp1^−/−^ BMDM showed equivalent sensitivity at 5 min, indicating that ablation of inflammasome components does not alter cell sensitivity to direct pore formation (Figure 5A). However, by 30 min, the extreme sensitivity of WT BMDM to SLO was revealed (Figure 5A). This extreme sensitivity was due to pyroptosis, since NLRP3^−/−^ and Casp1^−/−^ BMDM did not show similarly increased sensitivity (Figure 5A). Likewise, inhibition of NLRP3 inflammasome activation by KCl blocked pyroptosis (Figure 5B). Using this system, we tested the requirement for Dnase1L3 in pyroptosis. Unlike KCl, FCA only mildly impaired pyroptosis, leaving BMDM 2-fold less sensitive to SLO, compared to the 20-fold reduction observed with KCl (Figure 5B). Thus, Dnase1L3 inhibition can uncouple NLRP3-induced cytokine secretion from pyroptosis.

### Dnase1L3 Is Critical for *Salmonella*-Induced IL-1β and HMGB1 Release

**Figure 6:**
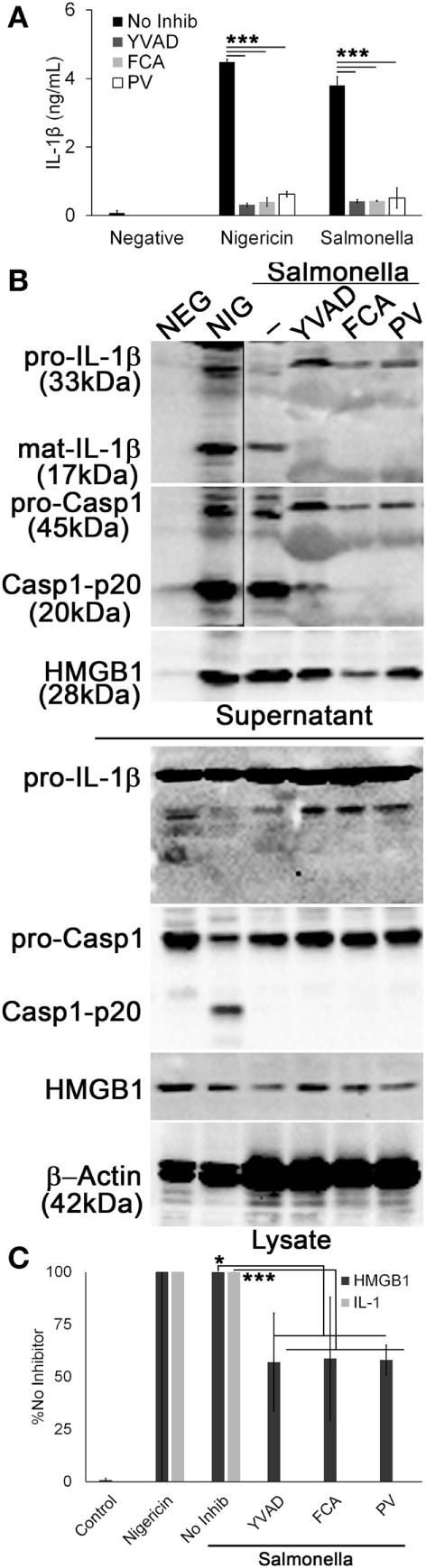
***Salmonella*-induced IL-1β release requires Dnase1L3 activity**. Lipopolysaccharide-primed BMDM were treated with or without 100 µM YVAD, 100 μM fmoc-d-cyclohexylalanine (FCA), and 50 µM pontacyl violet 6R (PV) followed by *Salmonella* (MOI 50) for 1 h or 20 µM nigericin for 30 min. **(A)** IL-1β in supernatants was detected by ELISA. **(B)** Proteins from trichloroacetic acid-precipitated supernatants and cell lysates were resolved by SDS-PAGE and transferred to nitrocellulose. High-mobility group box 1 protein (HMGB1), caspase-1 (Casp1), IL-1β, and β-actin were detected by western blot. **(C)** The levels of mature IL-1β in the supernatant were ratioed to pro-IL-1β levels in the cell lysates, or levels of secreted HMGB1 were ratioed to HMGB1 in the lysate, and all expressed as a percentage of B6 BMDM treated with *Salmonella* and no inhibitor. Graphs are mean ± SEM of four independent experiments, while immunoblots are representative of four independent experiments. *p < 0.05 or ***p < 0.001 for *Salmonella* with inhibitors compared to *Salmonella* no inhibitor.

Since Dnase1L3 activity was necessary for NLRP3-dependent Casp1 activation and IL-1β and HMGB1 release, we tested whether the requirement for Dnase1L3 is specific to NLRP3 or also required for other inflammasomes. We tested the NLRC4 inflammasome, since it can engage Casp1 directly (57). To test the role of Dnase1L3 during NLRC4 activation, we measured IL-1β release by ELISA from LPS-primed BMDM infected with *Salmonella enterica* typhimurium for 1 h. At this time point, *S*. typhimurium specifically triggers NLRC4-dependent, but NLRP3-independent inflammasome activation (30–32, 58). Under NLRC4-activating conditions, we found that FCA and PV reduced IL-1β release compared to the control (Figure 6A). Similarly, FCA and PV blocked mature IL-1β production and Casp1 cleavage following *S*. typhimurium infection (Figure 6B). Following quantitation of the western blots, we found that while IL-1β was potently blocked, HMGB1 egress was variably decreased following FCA or PV treatment (Figure 6C). This is consistent with the relatively mild ability of Dnase1L3 inhibitors to impair pyroptosis (Figure 5). Overall, we conclude that Dnase1L3 activity is also important for *Salmonella* typhimurium-induced Casp1 and IL-1β processing.

### RNAi of Dnase1L3 Blocks IL-1β Production and HMGB1 Release

**Figure 7:**
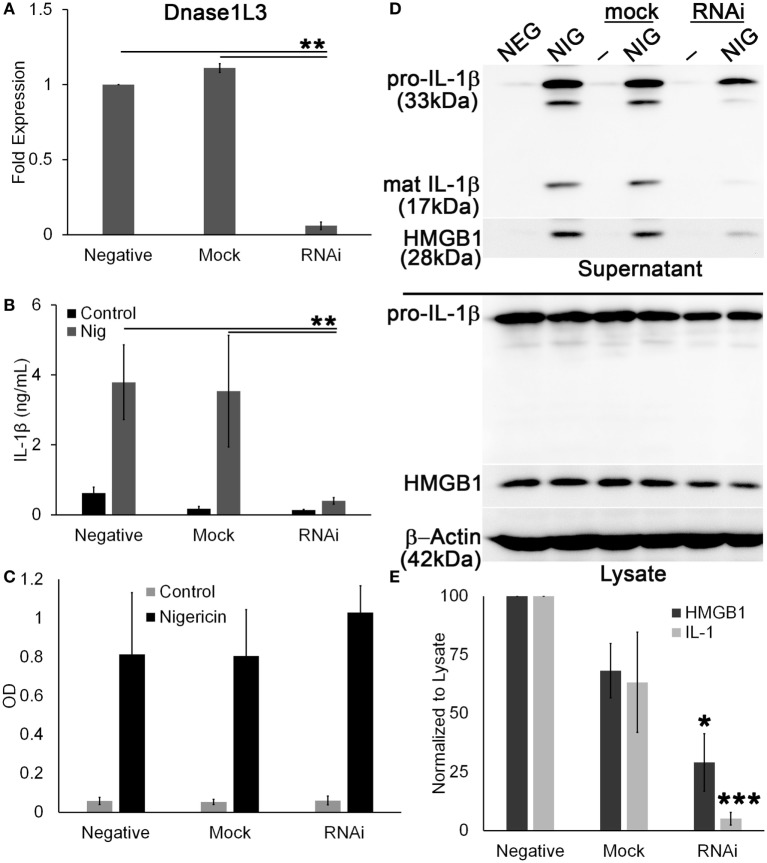
**Ablation of Dnase1L3 blocks IL-1β release**. THP1 cells were transfected with nothing, mock, or Dnase1L3 siRNA, then phorbol 12-myristate 13-acetate (PMA) differentiated for 2 days, and treated with 20 µM nigericin for 30 min. **(A)** Cells were then lysed in TriZOL, and total RNA was extracted from PMA-differentiated THP1 cells transfected with nothing, mock, or Dnase1L3 siRNA. Dnase1L3 expression was measured by qRT-PCR, using GAPDH as a control. Supernatants were used for ELISA **(B)**, LDH assay **(C)** or trichloroacetic acid precipitated **(D)**. Supernatants assayed for TNFα release were collected after PMA differentiation, but prior to nigericin treatment. Maximum TNFα release was always from non-transfected THP1 cells and was an average of 1.2 ng/mL. OD values for LDH assay represent OD_490_–OD_680_. **(D)** Cells were lysed in SDS sample buffer. Proteins were resolved by SDS-PAGE and transferred to nitrocellulose. High-mobility group box 1 protein (HMGB1), IL-1β, and β-actin were detected by western blot. **(E)** The levels of mature IL-1β in the supernatant were ratioed to pro-IL-1β levels in the cell lysates, or levels of secreted HMGB1 were ratioed to HMGB1 in the lysate, and expressed as a percentage of control, nigericin-treated cells. Graphs are mean ± SEM of 3 **(A–C)** or 12 **(E)** independent experiments, and immunoblots are representative of 12 independent experiments. *p < 0.05, **p < 0.01, ***p < 0.001, Dnase1L3 RNA interference (RNAi) compared to mock or negative.

Since pharmacological inhibitors can have off-target effects, and because FCA and PV are poorly characterized, we knocked down Dnase1L3 using RNAi in PMA-differentiated THP1 cells. PMA-differentiated THP1 cells are a human model system for inflammasome activation (59), so we used the model NLRP3 agonist nigericin to measure inflammasome activation. The mRNA knockdown efficiency of Dnase1L3 expression was typically ~90% (Figure 7A). We found that similar to the inhibitors, ablation of Dnase1L3 reduced IL-1β secretion by ELISA (Figure 7B). We tested whether pyroptosis was impaired by LDH release and found that this assay could not detect any significant change in pyroptosis between untransfected and Dnase1L3 siRNA-transfected cells (Figure 7C). Dnase1L3 RNAi reduced mature IL-1β processing compared to control cells (Figure 7D). Since there was some variability in cell numbers, we quantitated our western blots and ratioed IL-1β secretion and HMGB1 release to levels in the cytosol. We found that HMGB1 egress was ~75% blocked and IL-1β ~90% blocked compared to the control group (Figure 7E). Mock siRNA did not reduce IL-1β or HMGB1 release to similar levels, suggesting that the inhibition was specific to Dnase1L3 (Figure 7E). We conclude that Dnase1L3 is necessary for inflammasome-mediated cytokine release based on both genetic and inhibitor experiments.

### Dnase1L3 Promotes ASC Speck Formation

**Figure 8:**
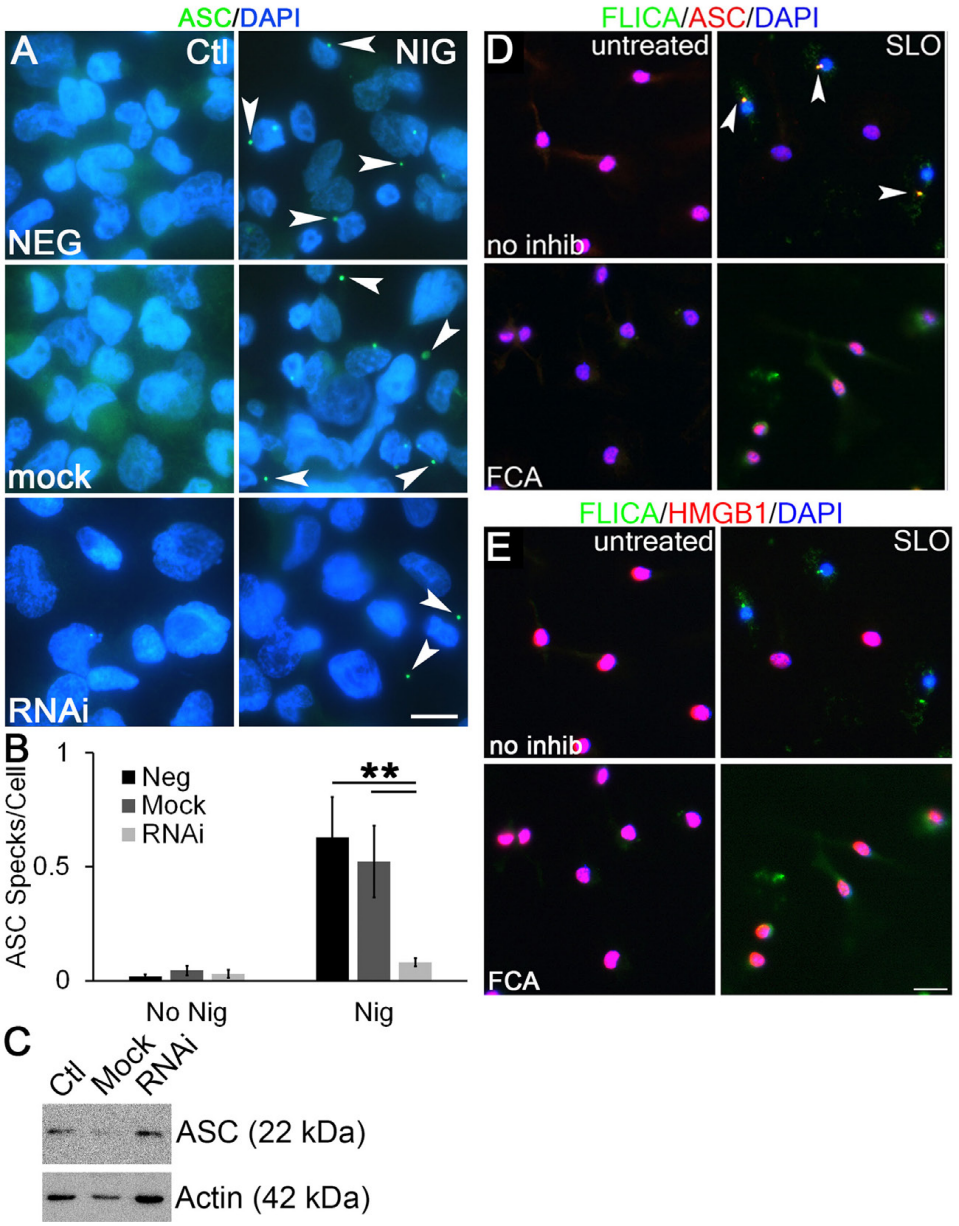
**Dnase1L3 promotes apoptosis-associated speck-like protein containing a caspase activation and recruitment domain (ASC) release from the nucleus**. **(A,B)** THP1 cells were transfected with nothing, mock, or Dnase1L3 siRNA, then phorbol 12-myristate 13-acetate (PMA) differentiated for 2 days, treated with or without 20 µM nigericin for 30 min, fixed, and stained for ASC (green) and DAPI (blue). **(A)** Representative micrographs show speck formation (arrowheads). **(B)** ASC specks were quantitated using ImageJ and normalized to the total number of cells. **(C)** THP1 cells were transfected with nothing, mock, or Dnase1L3 siRNA, then PMA differentiated for 2 days and lysed in SDS sample buffer. Proteins were resolved by SDS-PAGE, transferred to nitrocellulose, and probed with anti-ASC and anti-actin. **(D,E)** Lipopolysaccharide-primed BMDM were treated with fmoc-d-cyclohexylalanine (FCA) for 30 min followed by challenge with streptolysin O (SLO) in the presence of FLICA (green) for 1 h, fixed, and stained with anti-ASC [**(D)**, red] and anti-high-mobility group box 1 protein (HMGB1) Abs [**(E)**, red] and DAPI (blue). FLICA/ASC specks are shown by arrowheads. The graph is mean ± SEM of three independent experiments, immunoblot representative of four, and micrographs representative of three **(A)** or four **(D,E)** independent experiments. Scale bar = 10 µm **(A)** or 20 µm **(D,E)**. **p < 0.01.

To examine the mechanism through which Dnase1L3 potently regulates cytokine release, but only mildly affects pyroptosis, we examined ASC release and speck formation. Formation of ASC specks is required for efficient cytokine IL-1β secretion by both NLRP3 and NLRC4 inflammasomes (30, 32). In contrast, the absence of ASC speck formation reduces pyroptosis only twofold (32, 57). This is nearly identical to the phenotype we observed following Dnase1L3 blockade. We measured ASC speck formation in PMA-differentiated THP1 cells following Dnase1L3 RNAi and inflammasome activation. ASC speck formation induced by nigericin was abrogated following Dnase1L3 RNAi, but unperturbed in mock transfected and untransfected cells (Figures 8A,B). Under these conditions, we detected an average of one ASC speck for every two cells (Figure 8B). Following Dnase1L3 RNAi, this dropped to one ASC speck per 26 cells (Figure 8B). Finally, we tested whether ASC levels were perturbed by Dnase1L3 RNAi. We found that ASC levels relative to actin were not significantly altered by Dnase1L3 siRNA (Figure 8C). Overall, these data suggest that Dnase1L3 is necessary for ASC speck formation.

We next tested whether Dnase1L3 inhibition blocked ASC speck formation and Casp1 recruitment in primary BMDM using the more biologically relevant NLRP3 agonist, SLO. In LPS-primed cells, ASC localized to the nucleus (Figure 8D). Following inflammasome activation, we observed ASC recruitment to one to three specks in a subset of BMDM (Figure 8D). Not all cells contained specks, suggesting heterogeneity in inflammasome activation by SLO and consistent with our findings in THP1 cells with nigericin (Figure 8B). We tested whether these specks were sites of active inflammasome assembly by visualizing active Casp1 in these cells. Active Casp1 in SLO-treated BMDM cells was visualized using FLICA, a fluorogenic Casp1 substrate. Active Casp1 was present in the cytosol and colocalized with ASC in specks (Figure 8D). Only cells that formed specks were depleted of nuclear HMGB1, indicating secretion of HMGB1 (Figure 8E). We did not detect HMGB1 in inflammasome specks (Figure 8E). In contrast, BMDM pretreated with FCA showed only diffuse cytosolic Casp1 upon SLO exposure and no inflammasome speck formation (Figure 8D). HMGB1 generally remained in the nucleus of FCA-treated cells (Figure 8E). This suggested that FCA blocked recruitment of ASC and Casp1 to inflammasome specks. Thus, Dnase1L3 blockade uncouples IL-1β production from pyroptosis by preventing ASC nuclear egress and speck formation.

## Discussion

Dnase1L3 is an endonuclease associated with pediatric-onset SLE. Here, we ascribe a new function for Dnase1L3: regulation of inflammasome activation. Specifically, we found that Dnase1L3 inhibition blocks IL-1β and HMGB1 secretion following inflammasome activation by preventing ASC nuclear egress. In contrast, Dnase1L3 was not required for pyroptosis. These findings indicate that Dnase1L3 inhibitors functionally uncouple two major inflammasome effector functions: cytokine secretion and cell death. Regulation of inflammasome effector functions may also explain the delayed onset of immune activation compared to anti-dsDNA antibody production during the pediatric-onset SLE observed in Dnase1L3^−/−^ mice.

One potential caveat on our findings is the possibility of inhibitor off-target effects. There is relatively little known about either FCA (39) or PV (39, 60) as inhibitors of biological processes. These inhibitors may target more enzymes than Dnase1L3, and one of those enzymes may be responsible for the blockade we observe. This blockade appears specific to inflammasome activation, since these inhibitors block ASC speck formation without blocking TNFα release and without directly acting on Casp1. Regardless of molecular target, this phenotype warrants further characterization of these compounds for use as inflammasome inhibitors. However, our RNAi studies suggest that specific ablation of Dnase1L3 impairs inflammasome activation. While RNAi can also be plagued by off-target effects, we controlled for potential off-target effects through the use of control siRNAs and through the use of a pool of multiple siRNAs that target Dnase1L3 (51). Although validation of these results in the Dnase1L3^−/−^ mice (6, 41) would further bolster these results, knockout mice can also have compensatory mechanisms or unknown gene disruptions that can cloud interpretation of results. Overall, we believe that Dnase1L3 inhibition blocks inflammasome activation through blockade of ASC nuclear export.

Since we observe an intracellular phenotype for a secreted protein, our findings place new importance on resolving the controversy over Dnase1L3 localization. Although many studies with overexpression systems observed Dnase1L3 acting in the nucleus during apoptosis (40, 41, 47, 48), other studies clearly demonstrate that Dnase1L3 is secreted (6, 46, 61). We find that Dnase1L3 inhibition blocks ASC nuclear translocation, which is an intracellular event. We hypothesize that Dnase1L3 could be activated by cellular stressors present during inflammasome activation, such as reactive oxygen species or mitochondrial disruption. Whether this promotes endocytosis of Dnase1L3, alters Dnase1L3 translation, or promotes relocalization of Dnase1L3 remains to be determined, although the kinetics of the interaction suggest that an intracellular pool of Dnase1L3 is needed. Our findings open new avenues of research into dissecting how Dnase1L3 is activated during inflammasome activation, and how a protein that is primarily secreted is able to act in the nucleus.

Dnase1L3 inhibition blocked IL-1β release following both NLRP3 and NLRC4 inflammasome activation. This result is consistent with findings that NLRC4 requires ASC for full inflammasome activation and maximal IL-1β release (30, 32). The twofold decrease in pyroptosis observed here is consistent with other studies examining the role of speck formation during pyroptosis (30). The partial inhibition of pyroptosis also accounts for the limited HMGB1 release we observe during Dnase1L3 inhibition. Dnase1L3 inhibition does not block the release of HMGB1 from lysed cells, such as pyroptotic or necrotic cells, but does reduce HMGB1 release stimulated by Casp1 cleavage and ASC translocation. Mechanistically, we have identified a role for Dnase1L3 in ASC release from nuclear or perinuclear stores. Although there are reports of cytosolic ASC in resting immune cells (23), this ASC was transfected, and the cytosolic ASC was either inactive and/or of insufficient concentration to promote pyroptosome assembly. Dnase1L3 activity appears to promote ASC nuclear export and speck formation in both human cell lines and primary murine BMDM. Whether this only increases the concentration of ASC or also permits phosphorylation remains to be determined. Similarly, whether Dnase1L3 directly interacts with ASC, alters IKKα, activates a kinase that phosphorylates ASC, or simply cleaves DNA remains to be determined. Since FCA blocks Dnase1L3 nuclease activity (39), one possibility is that Dnase1L3 nuclease activity is needed to release DNA-binding proteins that participate in IL-1β processing. For example, Dnase1L3 could potentially cleave chromosomal DNA, which would liberate proteins bound to DNA. These proteins could promote ASC egress from the nucleus and subsequent inflammasome activation. In contrast, we do not expect Casp11 or Gasdermin D activation to be significantly affected by Dnase1L3 inhibition, since pyroptosis is only mildly impaired at best. Dnase1L3 inhibition uncouples cytokine secretion from pyroptosis. This uncoupling should allow future studies to determine the molecular mechanisms that specifically initiate pyroptosis as well as the machinery required for cytokine secretion.

For diseases caused by excess of inflammasome-released cytokines, FCA or PV may show potential as novel therapeutics. Although long-term Dnase1L3 inhibition may lead to drug-induced SLE, restoration of Dnase activity reverses SLE phenotypes (6). This suggests that the risks may be outweighed by the potential benefits of blocking both HMGB1 and IL-1β. Interdiction of HMGB1 activity through neutralizing Abs or soluble Receptor for Advanced Glycation Endproducts is protective in multiple systems, including sepsis, endotoxemia, traumatic injury, cancer, colitis, arthritis, and infection (36). Similarly, neutralizing IL-1β activity through the use of the IL-1 receptor antagonist is protective in multiple systems, including arthritis, diabetes, metabolic syndrome, psoriasis, periodic fevers, and gout among others (62). However, these treatments rely on blocking the activity of secreted IL-1β and HMGB1. Blockade of HMGB1 and IL-1β release using Dnase1L3 inhibitors offers a target for therapy that acts on two critical pro-inflammatory proteins and perhaps offers greater effectiveness than monotherapy.

Finally, our findings provide a mechanistic explanation for a surprising phenotype observed in Dnase1L3^−/−^ mice. These mice show immune activation defects and poor production of autoantibodies aside from anti-dsDNA antibodies (6). Previously, it was not clear why anti-dsDNA antibodies, but not other antinuclear antibodies or the typical immune response associated with SLE, rapidly occur if the only defect in Dnase1L3^−/−^ organisms was failure to digest dsDNA in apoptotic bodies. Our finding that Dnase1L3 is needed for inflammasome activation provides one explanation for the observed delayed onset of immunity between anti-dsDNA antibodies and other autoantibodies and immune activation. The NLRP3 inflammasome mediates the adjuvant effect for many adjuvants, including alum (63, 64). Impairment of inflammasome activation prevents priming of anti-ovalbumin responses (63), suggesting that blockade of the inflammasome may prevent antibody formation. Since Dnase1L3 inhibition blocks cytokine secretion during inflammasome activation, this effect may account for the delayed onset of immunity in Dnase1L3^−/−^ mice. Future work is needed to test these ideas, especially since in the B6/*lpr* autoimmune model, NLRP3 and ASC can play a protective role against autoimmunity (65), which is the opposite of what we predict here. However, this mouse model suffers from lymphoproliferative disease not present in human SLE and their observation was based on global deletion of NLRP3 or ASC and ascribed to TGF-β signaling acting independently of the inflammasome (65). In addition, autoantibodies, especially anti-dsDNA antibodies, can be primed by TLR stimulation, such as by TLR9. This could explain the rapid production of anti-dsDNA antibodies in the absence of other antinuclear antibodies, similar to the situation observed in Dnase2a/STING double knockout mice (9). Thus, we propose one possible mechanism for delayed onset of immunity in the murine model of pediatric-onset SLE.

## Ethics Statement

This study was carried out in accordance with the recommendations of local, state and federal guidelines as overseen by the Texas Tech University Animal Care and Use Committee. The protocol was approved by the Texas Tech University Animal Care and Use Committee.

## Author Contributions

GS, KA, WW, and PK performed experiments. RS and PK designed the experiments. GS, RS, and PK prepared the manuscript. GS, KA, WW, RS, and PK analyzed data, critically revised the manuscript, and approved the final manuscript.

## Conflict of Interest Statement

The authors declare that the research was conducted in the absence of any commercial or financial relationships that could be construed as a potential conflict of interest.

## Abbreviations

anti-dsDNA, anti-double-stranded DNA; ASC, apoptosis-associated speck-like protein containing a CARD; ATA, aurintricarboxylic acid; BMDM, bone marrow-derived macrophages; CARD, caspase activation and recruitment domain; Casp1, caspase-1; D10, DMEM supplemented with 10% FCS and 1× l-glutamine; FCA, fmoc-d-cyclohexylalanine; FLICA, Fam-YVAD-fmk; HMGB1, high-mobility group box 1 protein; HU, hemolytic units; NLR, Nod-like receptor; NLRC4, NLR family, CARD domain containing 4; NLRP3, NLR family, pyrin domain containing 3; PFT, pore-forming toxin; PI, propidium iodide; PV, pontacyl violet 6R; RAGE, Receptor for Advanced Glycation Endproducts; RNAi, RNA interference; SLE, systemic lupus erythematosus; SLO, streptolysin O; WT, wild type.

## References

[1] Relle M Weinmann-Menke J Scorletti E Cavagna L Schwarting A. Genetics and novel aspects of therapies in systemic lupus erythematosus. Autoimmun Rev (2015) 14(11):1005–18. 10.1016/j.autrev.2015.07.003 26164648

[2] Rekvig OP van der Vlag J Seredkina N Review: antinucleosome antibodies: a critical reflection on their specificities and diagnostic impact. Arthritis Rheumatol (2014) 66(5):1061–9. 10.1002/art.38365 24470458

[3] Choi J Kim ST Craft J. The pathogenesis of systemic lupus erythematosus – an update. Curr Opin Immunol (2012) 24(6):651–7. 10.1016/j.coi.2012.10.004 23131610 PMC3508331

[4] Al-Mayouf SM Al Sonbul A. Influence of gender and age of onset on the outcome in children with systemic lupus erythematosus. Clin Rheumatol (2008) 27(9):1159–62. 10.1007/s10067-008-0887-z 18421546

[5] Al-Mayouf SM Sunker A Abdwani R Abrawi SA Almurshedi F Alhashmi N Loss-of-function variant in DNASE1L3 causes a familial form of systemic lupus erythematosus. Nat Genet (2011) 43(12):1186–8. 10.1038/ng.975 22019780

[6] Sisirak V Sally B D’Agati V Martinez-Ortiz W Ozcakar ZB David J Digestion of chromatin in apoptotic cell microparticles prevents autoimmunity. Cell (2016) 166(1):88–101. 10.1016/j.cell.2016.05.034 27293190 PMC5030815

[7] Ozcakar ZB Foster J II Diaz-Horta O Kasapcopur O Fan YS Yalcinkaya F DNASE1L3 mutations in hypocomplementemic urticarial vasculitis syndrome. Arthritis Rheum (2013) 65(8):2183–9. 10.1002/art.38010 23666765

[8] Ueki M Kimura-Kataoka K Takeshita H Fujihara J Iida R Sano R Evaluation of all non-synonymous single nucleotide polymorphisms (SNPs) in the genes encoding human deoxyribonuclease I and I-like 3 as a functional SNP potentially implicated in autoimmunity. FEBS J (2014) 281(1):376–90. 10.1111/febs.12608 24206041

[9] Baum R Sharma S Carpenter S Li QZ Busto P Fitzgerald KA Cutting edge: AIM2 and endosomal TLRs differentially regulate arthritis and autoantibody production in DNase II-deficient mice. J Immunol (2015) 194(3):873–7. 10.4049/jimmunol.1402573 25548216 PMC4299698

[10] Fontes FL Pinheiro DM Oliveira AH Oliveira RK Lajus TB Agnez-Lima LF. Role of DNA repair in host immune response and inflammation. Mutat Res Rev Mutat Res (2015) 763:246–57. 10.1016/j.mrrev.2014.11.004 25795123

[11] Fernandes-Alnemri T Yu JW Datta P Wu J Alnemri ES. AIM2 activates the inflammasome and cell death in response to cytoplasmic DNA. Nature (2009) 458(7237):509–13. 10.1038/nature07710 19158676 PMC2862225

[12] Kawane K Tanaka H Kitahara Y Shimaoka S Nagata S. Cytokine-dependent but acquired immunity-independent arthritis caused by DNA escaped from degradation. Proc Natl Acad Sci U S A (2010) 107(45):19432–7. 10.1073/pnas.1010603107 20974942 PMC2984163

[13] Ahn J Gutman D Saijo S Barber GN. STING manifests self DNA-dependent inflammatory disease. Proc Natl Acad Sci U S A (2012) 109(47):19386–91. 10.1073/pnas.1215006109 23132945 PMC3511090

[14] Atianand MK Fitzgerald KA Molecular basis of DNA recognition in the immune system. J Immunol (2013) 190(5):1911–8. 10.4049/jimmunol.1203162 23417527 PMC3660856

[15] Kanneganti TD Central roles of NLRs and inflammasomes in viral infection. Nat Rev Immunol (2010) 10(10):688–98. 10.1038/nri2851 20847744 PMC3909537

[16] Franchi L Munoz-Planillo R Nunez G. Sensing and reacting to microbes through the inflammasomes. Nat Immunol (2012) 13(4):325–32. 10.1038/ni.2231 22430785 PMC3449002

[17] Keyel PA. How is inflammation initiated? Individual influences of IL-1, IL-18 and HMGB1. Cytokine (2014) 69(1):136–45. 10.1016/j.cyto.2014.03.007 24746243

[18] Harder J Franchi L Munoz-Planillo R Park JH Reimer T Nunez G. Activation of the Nlrp3 inflammasome by *Streptococcus pyogenes* requires streptolysin O and NF-kappa B activation but proceeds independently of TLR signaling and P2X7 receptor. J Immunol (2009) 183(9):5823–9. 10.4049/jimmunol.0900444 19812205 PMC2765568

[19] Keyel PA Roth R Yokoyama WM Heuser JE Salter RD. Reduction of streptolysin O (SLO) pore-forming activity enhances inflammasome activation. Toxins (2013) 5(6):1105–18. 10.3390/toxins5061105 23744055 PMC3717772

[20] Zhao Y Yang J Shi J Gong YN Lu Q Xu H The NLRC4 inflammasome receptors for bacterial flagellin and type III secretion apparatus. Nature (2011) 477(7366):596–600. 10.1038/nature10510 21918512

[21] Bryan NB Dorfleutner A Rojanasakul Y Stehlik C. Activation of inflammasomes requires intracellular redistribution of the apoptotic speck-like protein containing a caspase recruitment domain. J Immunol (2009) 182(5):3173–82. 10.4049/jimmunol.0802367 19234215 PMC2652671

[22] Martin BN Wang C Willette-Brown J Herjan T Gulen MF Zhou H IKKalpha negatively regulates ASC-dependent inflammasome activation. Nat Commun (2014) 5:4977 10.1038/ncomms5977 25266676 PMC4298287

[23] Fernandes-Alnemri T Wu J Yu JW Datta P Miller B Jankowski W The pyroptosome: a supramolecular assembly of ASC dimers mediating inflammatory cell death via caspase-1 activation. Cell Death Differ (2007) 14(9):1590–604. 10.1038/sj.cdd.4402194 17599095 PMC3345951

[24] Cai X Chen J Xu H Liu S Jiang QX Halfmann R Prion-like polymerization underlies signal transduction in antiviral immune defense and inflammasome activation. Cell (2014) 156(6):1207–22. 10.1016/j.cell.2014.01.063 24630723 PMC4034535

[25] Lu A Magupalli VG Ruan J Yin Q Atianand MK Vos MR Unified polymerization mechanism for the assembly of ASC-dependent inflammasomes. Cell (2014) 156(6):1193–206. 10.1016/j.cell.2014.02.008 24630722 PMC4000066

[26] Miao EA Rajan JV Aderem A. Caspase-1-induced pyroptotic cell death. Immunol Rev (2011) 243(1):206–14. 10.1111/j.1600-065X.2011.01044.x 21884178 PMC3609431

[27] Kayagaki N Stowe IB Lee BL O’Rourke K Anderson K Warming S Caspase-11 cleaves gasdermin D for non-canonical inflammasome signalling. Nature (2015) 526(7575):666–71. 10.1038/nature15541 26375259

[28] Shi J Zhao Y Wang K Shi X Wang Y Huang H Cleavage of GSDMD by inflammatory caspases determines pyroptotic cell death. Nature (2015) 526(7575):660–5. 10.1038/nature15514 26375003

[29] Ding J Wang K Liu W She Y Sun Q Shi J Pore-forming activity and structural autoinhibition of the gasdermin family. Nature (2016) 535(7610):111–6. 10.1038/nature18590 27281216

[30] Broz P Newton K Lamkanfi M Mariathasan S Dixit VM Monack DM Redundant roles for inflammasome receptors NLRP3 and NLRC4 in host defense against *Salmonella*. J Exp Med (2010) 207(8):1745–55. 10.1084/jem.20100257 20603313 PMC2916133

[31] Qu Y Misaghi S Newton K Maltzman A Izrael-Tomasevic A Arnott D NLRP3 recruitment by NLRC4 during *Salmonella* infection. J Exp Med (2016) 213(6):877–85. 10.1084/jem.20132234 27139490 PMC4886354

[32] Mariathasan S Newton K Monack DM Vucic D French DM Lee WP Differential activation of the inflammasome by caspase-1 adaptors ASC and Ipaf. Nature (2004) 430(6996):213–8. 10.1038/nature02664 15190255

[33] Heid ME Keyel PA Kamga C Shiva S Watkins SC Salter RD. Mitochondrial reactive oxygen species induces NLRP3-dependent lysosomal damage and inflammasome activation. J Immunol (2013) 191(10):5230–8. 10.4049/jimmunol.1301490 24089192 PMC3833073

[34] Willingham SB Allen IC Bergstralh DT Brickey WJ Huang MT Taxman DJ NLRP3 (NALP3, Cryopyrin) facilitates in vivo caspase-1 activation, necrosis, and HMGB1 release via inflammasome-dependent and -independent pathways. J Immunol (2009) 183(3):2008–15. 10.4049/jimmunol.0900138 19587006 PMC3652593

[35] Lamkanfi M Sarkar A Vande Walle L Vitari AC Amer AO Wewers MD Inflammasome-dependent release of the alarmin HMGB1 in endotoxemia. J Immunol (2010) 185(7):4385–92. 10.4049/jimmunol.1000803 20802146 PMC3428148

[36] Lotze MT Tracey KJ. High-mobility group box 1 protein (HMGB1): nuclear weapon in the immune arsenal. Nat Rev Immunol (2005) 5(4):331–42. 10.1038/nri1594 15803152

[37] Kim JH Kim SJ Lee IS Lee MS Uematsu S Akira S Bacterial endotoxin induces the release of high mobility group box 1 via the IFN-beta signaling pathway. J Immunol (2009) 182(4):2458–66. 10.4049/jimmunol.0801364 19201901

[38] Wang H Bloom O Zhang M Vishnubhakat JM Ombrellino M Che J HMG-1 as a late mediator of endotoxin lethality in mice. Science (1999) 285(5425):248–51. 10.1126/science.285.5425.248 10398600

[39] Yamada Y Fujii T Ishijima R Tachibana H Yokoue N Takasawa R DR396, an apoptotic DNase gamma inhibitor, attenuates high mobility group box 1 release from apoptotic cells. Bioorg Med Chem (2011) 19(1):168–71. 10.1016/j.bmc.2010.11.037 21167721

[40] Shiokawa D Tanuma S. Characterization of human DNase I family endonucleases and activation of DNase gamma during apoptosis. Biochemistry (2001) 40(1):143–52. 10.1021/bi001041a 11141064

[41] Mizuta R Araki S Furukawa M Furukawa Y Ebara S Shiokawa D DNase gamma is the effector endonuclease for internucleosomal DNA fragmentation in necrosis. PLoS One (2013) 8(12):e80223 10.1371/journal.pone.0080223 24312463 PMC3846476

[42] Wilber A Lu M Schneider MC. Deoxyribonuclease I-like III is an inducible macrophage barrier to liposomal transfection. Mol Ther (2002) 6(1):35–42. 10.1006/mthe.2002.0625 12095301

[43] Shiokawa D Ohyama H Yamada T Takahashi K Tanuma S. Identification of an endonuclease responsible for apoptosis in rat thymocytes. Eur J Biochem (1994) 226(1):23–30. 10.1111/j.1432-1033.1994.tb20022.x 7957253

[44] Liu QY Pandey S Singh RK Lin W Ribecco M Borowy-Borowski H DNaseY: a rat DNaseI-like gene coding for a constitutively expressed chromatin-bound endonuclease. Biochemistry (1998) 37(28):10134–43. 10.1021/bi9800597bi9800597 9665719

[45] Shiokawa D Shika Y Tanuma S. Identification of two functional nuclear localization signals in DNase gamma and their roles in its apoptotic DNase activity. Biochem J (2003) 376(Pt 2):377–81. 10.1042/BJ20030820 12943533 PMC1223774

[46] Napirei M Wulf S Eulitz D Mannherz HG Kloeckl T. Comparative characterization of rat deoxyribonuclease 1 (Dnase1) and murine deoxyribonuclease 1-like 3 (Dnase1l3). Biochem J (2005) 389(Pt 2):355–64. 10.1042/BJ20042124 15796714 PMC1175112

[47] Boulares AH Zoltoski AJ Sherif ZA Yakovlev AG Smulson ME. The Poly(ADP-ribose) polymerase-1-regulated endonuclease DNAS1L3 is required for etoposide-induced internucleosomal DNA fragmentation and increases etoposide cytotoxicity in transfected osteosarcoma cells. Cancer Res (2002) 62(15):4439–44. 12154052

[48] Boulares AH Ren T. Mechanism of acetaminophen-induced apoptosis in cultured cells: roles of caspase-3, DNA fragmentation factor, and the Ca2+ and Mg2+ endonuclease DNAS1L3. Basic Clin Pharmacol Toxicol (2004) 94(1):19–29. 10.1111/j.1742-7843.2004.pto_940105.x 14725611

[49] Keyel PA Heid ME Watkins SC Salter RD. Visualization of bacterial toxin induced responses using live cell fluorescence microscopy. J Vis Exp (2012) 68:e4227. 10.3791/4227 23052609 PMC3490310

[50] Keyel PA Romero M Wu W Kwak DH Zhu Q Liu X Methylthioadenosine reprograms macrophage activation through adenosine receptor stimulation. PLoS One (2014) 9(8):e104210. 10.1371/journal.pone.0104210 25117662 PMC4130577

[51] Jackson AL Linsley PS. Recognizing and avoiding siRNA off-target effects for target identification and therapeutic application. Nat Rev Drug Discov (2010) 9(1):57–67. 10.1038/nrd3010 20043028

[52] Agrawal N Dasaradhi PV Mohmmed A Malhotra P Bhatnagar RK Mukherjee SK. RNA interference: biology, mechanism, and applications. Microbiol Mol Biol Rev (2003) 67(4):657–85. 10.1128/MMBR.67.4.657-685.2003 14665679 PMC309050

[53] Rasband WS ImageJ. Bethesda, MD: U.S. National Institutes of Health (1997–2016).

[54] Gong YN Wang X Wang J Yang Z Li S Yang J Chemical probing reveals insights into the signaling mechanism of inflammasome activation. Cell Res (2010) 20(12):1289–305. 10.1038/cr.2010.135 20856264

[55] Imamura R Wang Y Kinoshita T Suzuki M Noda T Sagara J Anti-inflammatory activity of PYNOD and its mechanism in humans and mice. J Immunol (2010) 184(10):5874–84. 10.4049/jimmunol.0900779 20393137

[56] Timmer AM Timmer JC Pence MA Hsu LC Ghochani M Frey TG Streptolysin O promotes group A *Streptococcus* immune evasion by accelerated macrophage apoptosis. J Biol Chem (2009) 284(2):862–71. 10.1074/jbc.M804632200 19001420 PMC2613605

[57] Broz P von Moltke J Jones JW Vance RE Monack DM Differential requirement for Caspase-1 autoproteolysis in pathogen-induced cell death and cytokine processing. Cell Host Microbe (2010) 8(6):471–83. 10.1016/j.chom.2010.11.007 21147462 PMC3016200

[58] Miao EA Alpuche-Aranda CM Dors M Clark AE Bader MW Miller SI Cytoplasmic flagellin activates caspase-1 and secretion of interleukin 1beta via Ipaf. Nat Immunol (2006) 7(6):569–75. 10.1038/ni1344 16648853

[59] Martinon F Burns K Tschopp J. The inflammasome: a molecular platform triggering activation of inflammatory caspases and processing of proIL-beta. Mol Cell (2002) 10(2):417–26. 10.1016/S1097-2765(02)00599-3 12191486

[60] Wood ML Green JA Studies on textile dyes for biological staining. I. Pontacyl blue black SX, pontacyl violet 6R and luxol fast yellow TN. Stain Technol (1958) 33(6):279–81. 10.3109/10520295809111863 13603079

[61] Wilber A O’Connor TP Lu ML Karimi A Schneider MC Dnase1l3 deficiency in lupus-prone MRL and NZB/W F1 mice. Clin Exp Immunol (2003) 134(1):46–52. 10.1046/j.1365-2249.2003.02267.x 12974753 PMC1808827

[62] Dinarello CA Simon A van der Meer JW. Treating inflammation by blocking interleukin-1 in a broad spectrum of diseases. Nat Rev Drug Discov (2012) 11(8):633–52. 10.1038/nrd3800 22850787 PMC3644509

[63] Kool M Petrilli V De Smedt T Rolaz A Hammad H van Nimwegen M Cutting edge: alum adjuvant stimulates inflammatory dendritic cells through activation of the NALP3 inflammasome. J Immunol (2008) 181(6):3755–9. 10.4049/jimmunol.181.6.3755 18768827

[64] Li H Willingham SB Ting JP Re F. Cutting edge: inflammasome activation by alum and alum’s adjuvant effect are mediated by NLRP3. J Immunol (2008) 181(1):17–21. 10.4049/jimmunol.181.1.17 18566365 PMC2587213

[65] Lech M Lorenz G Kulkarni OP Grosser MO Stigrot N Darisipudi MN NLRP3 and ASC suppress lupus-like autoimmunity by driving the immunosuppressive effects of TGF-beta receptor signalling. Ann Rheum Dis (2015) 74(12):2224–35. 10.1136/annrheumdis-2014-205496 25135254

